# A New Prognostic Index PDPI for the Risk of Pneumonia Among Patients With Diabetes

**DOI:** 10.3389/fcimb.2021.723666

**Published:** 2021-09-06

**Authors:** Lingxi Guo, Yanyan Song, Ni Li, Binbin Qin, Bin Hu, Huahua Yi, Jingwen Huang, Bing Liu, Liping Yu, Yi Huang, Min Zhou, Jieming Qu

**Affiliations:** ^1^Department of Pulmonary and Critical Care Medicine, Ruijin Hospital, Shanghai Jiao Tong University School of Medicine, Shanghai, China; ^2^Institute of Respiratory Diseases, Shanghai Jiao Tong University School of Medicine, Shanghai, China; ^3^Shanghai Key Laboratory of Emergency Prevention, Diagnosis and Treatment of Respiratory Infectious Diseases, Shanghai, China; ^4^Department of Biostatistics, Clinical Research Institute, Shanghai Jiaotong University School of Medicine, Shanghai, China; ^5^Department of Respiratory Disease, The People’s Hospital of Putuo District, Shanghai, China; ^6^Department of Respiratory Disease, Huangpu Branch of the Ninth People’s Hospital Affiliated to Shanghai Jiao Tong University School of Medicine, Shanghai, China; ^7^Department of Respiratory Disease, Xuhui District Central Hospital, Shanghai, China; ^8^Department of Endocrinology, China-Japan Friendship Hospital, Beijing, China; ^9^Department of Respiratory and Critical Care Medicine, Navy Medical University Pulmonary and Critical Care Medicine, Shanghai, China

**Keywords:** diabetes, pneumonia, predicting score, risk factor, nomogram

## Abstract

**Objective:**

Risk factors for the development of pneumonia among patients with diabetes mellitus are unclear. The aim of our study was to elucidate the potential risk factors and attempt to predict the probability of pneumonia based on the history of diabetes.

**Methods:**

We performed a population-based, prospective multicenter cohort study of 1,043 adult patients with diabetes in China during 2017–2019. Demographic information, comorbidities, or laboratory examinations were collected.

**Results:**

The study included 417 diabetic patients with pneumonia and 626 no-pneumonia-onset diabetic patients. The predictive risk factors were chosen on the basis of a multivariate logistic regression model to predict pneumonia among patients with diabetes including male sex [odds ratio (OR) = 1.72, 95% confidence interval (CI): 1.27–2.33, p < 0.001], age ≥ 75 years (OR = 2.31, 95% CI: 1.61–3.31, p < 0.001), body mass index < 25 (OR = 2.59, 95% CI: 1.92–3.50, p < 0.001), chronic obstructive pulmonary disease (OR = 6.58, 95% CI: 2.09–20.7, p = 0.001), hypertension (OR = 4.27, 95% CI: 3.12–5.85, p < 0.001), coronary heart disease (OR = 2.98, 95% CI: 1.61–5.52, p < 0.001), renal failure (OR = 1.82, 95% CI: 1.002–3.29, p = 0.049), cancer (OR = 3.57, 95% CI: 1.80–7.06, p < 0.001), use of insulin (OR = 2.28, 95% CI: 1.60–3.25, p < 0.001), and hemoglobin A1c ≥ 9% (OR = 2.70, 95% CI: 1.89–3.85, p < 0.001). A predictive nomogram was established. This model showed c-statistics of 0.811, and sensitivity and specificity were 0.717 and 0.780, respectively, under cut-off of 125 score.

**Conclusion:**

We designed a clinically predictive tool for assessing the risk of pneumonia among adult patients with diabetes. This tool stratifies patients into relevant risk categories and may provide a basis for individually tailored intervention for the purpose of early prevention.

## Introduction

Pneumonia, either community-acquired pneumonia or hospital-acquired pneumonia, is the sixth leading cause of death; its incidence has increased in the past years, especially in high-and middle-income countries (Dagenais et al., 2019). In the absence of appropriate antibiotic treatment, the mortality rate among patients with ventilator-associated pneumonia may reach 70% (Vardakas et al., 2007). Efforts should be made to identify the potential factors associated with the onset of pneumonia.

Diabetes mellitus (DM) increases vulnerability to pneumonia; its prevalence ranges 6–25% (Falguera et al., 2005; Falcone et al., 2016), rendering patients with diabetes more susceptible to pneumonia (Kornum et al., 2008). The rates of hospitalization due to pneumonia among patients with type 2 diabetes have been noticeably increasing (based on data from 2004 to 2013) (Lopez-de-Andres et al., 2017). A previous study also demonstrated an elevated risk of pneumonia in patients hospitalized with diabetes, which remained unchanged across different time intervals (Shah and Hux, 2003). Hyperglycemia results in impairment of immune pathways, including neutrophil migration and antibody production (Jafar et al., 2016). Furthermore, diabetes influences the onset and outcomes of specific infections (e.g., pneumococcal pneumonia), while the risk of admission due to pneumonia remained high despite a national policy recommending routine pneumococcal immunization for diabetic patients in the United Kingdom (Seminog and Goldacre, 2013). Therefore, the socioeconomic burden of DM is large, especially for the emergency department, and increasing at a rapid rate in the Unites States of America (Korbel and Spencer, 2015). Clinicians should be aware of the high risk of pneumonia among patients with diabetes and pay more attention to potential preventions.

This was a 2-year prospective cohort study designed to evaluate the potential risk factors associated with the occurrence of pneumonia in patients with history of DM. Our aim was to elucidate the potential risk factors and set up a predictive model for the probability of pneumonia based on the diagnosis of diabetes.

## Research Design and Methods

### Study Design and Data Collection

The present study was based on a population-based, prospective, cohort study involving adult patients with diabetes. It was conducted as a prospective follow-up study with the aim of investigating the influence of multiple baseline variables on the risk of pneumonia in diabetes. This study was approved by the Ruijin Hospital Ethics Committee in Shanghai (No.2017-205) and registered in the clinicaltrials.gov database (NCT 03617393).

The typical treatment for most patients with diabetes is regular outpatient follow-up. Therefore, we conducted a community-based epidemiological survey of residents in China. Community residents who attended the community health screening activities were invited to participate in this follow-up study. We consecutively recruited residents with a history of DM who agreed to participate in this study from August 2017 to May 2019. They were asked to complete a structured questionnaire focusing on clinical characteristics, including demographic features, presence of chronic disease, family history, and glucose control. Patients were included according to the following inclusion criteria: 1) age ≥ 18 years; 2) clear diagnosis of diabetes; and 3) complete baseline information available. Patients with a recent diagnosis of pneumonia (within 3 months prior to study enrollment) were excluded (n = 43) in case of the potential bias. Meanwhile, clinical information of patients from this cohort who were diagnosed with pneumonia during the follow-up, according to the 2009 Infectious Diseases Society of American (IDSA)/American Thoracic Society (ATS) guideline, was recorded. During hospitalization for these patients, glycemic monitoring and measurement, including fasting blood glucose (FBG), 2-h postprandial blood glucose (PBG), and hemoglobin A1c (HbA1c) were assayed using venous blood samples on admission. We randomly matched diabetic patients without pneumonia at the time of follow-up in this study with patients diagnosed with pneumonia by 1:1.5 to form a no-pneumonia-onset group.

### Statistical Methods

Subjects were compared according to the onset of pneumonia or simple diabetic patients without respiratory infection. Univariate analysis was initially used to compare risk factors for pneumonia independently among patients with diabetes. Proportions or means with standard deviation were used to characterize the patient sample. Continuous variables were compared using t-tests or one-way ANOVA while χ^2^ or Fisher’s exact tests were used for categorical dependent data analysis, as appropriate.

Continuous variables were categorized and retained for multivariate testing. Cut-off points were identified following Youden’s index of the receiver operating characteristic curve or a clinically relevant cut-off. All univariate predictors with p < 0.10 were regarded as potential risk factors (Wang et al., 2017) and included in the multivariate regression analysis against overall mortality reduced through a backward elimination procedure (conditional likelihood ratio test and elimination if p ≥ 0.05).

Collinearity diagnosis was performed by calculating the variance inflation factor (VIF) of each predictor. Variables with VIF ≥ 2.0 were excluded from the multivariate analysis. Correlation coefficient matrix analysis was also conducted to clarify the degree of correlation between predictors.

The data of 1,043 patients were randomly assigned into two complementary subsets: the training set of 834 patients (80%) was used to establish the model; and the testing set of 209 patients (20%) was used to validate the analysis. The prognostic index of patients with diabetes was calculated based on the results of the multivariate analysis, in which odds ratios (ORs) and 95% confidence intervals (CIs) were calculated for each predictor. The performance of the score was assessed by measuring the area under the receiver operating characteristic curve (AUROC), while sensitivity and specificity were also calculated.

Internal validation was performed by applying the 5,000 bootstrap re-sampling technique. Cross-validation was assessed by calculating the AUROC of the testing set. The accuracy of the model was tested using a calibration plot referring to the agreement between observed outcomes and predictions (Barili et al., 2013). The perfect calibrated prediction stays on the 45-degree line, while a curve below and above the diagonal reflects overestimation and underestimation, respectively. The Hosmer–Lemeshow test was also performed, and a p > 0.05 indicated a good fit for the model.

Prognostic groups were defined by categorizing the prognostic score of the final logistic regression model. The optimal cut-off points were determined by minimizing the p-value relating the prognostic score to outcome according to the “minimal P value approach,” (Altman et al., 1994) and p-values were adjusted for multiple testing using the Bonferroni method. The goal was to define four risk groups for practical reasons referring to the clinical application of the prognostic index; none of the groups comprised >50% of the patients.

The validity of the model was retested using 10-fold cross-validation in the derivation cohort, which was divided randomly into 10 subsamples. A logistic regression model was developed using nine subsamples, and the score was then calculated using the remaining (held-out) subsample. This process was repeated for each of 10 loops of cross-validation so that in the end each patient had a score, with which overall (cross-validated) association and inference was tested.

Statistical analysis was performed using the SPSS version 22.0 and R 3.5.0. All tests were two-sided, and a p < 0.05 denoted statistical significance.

## Results

### Patient Characteristics

**Figure 1:**
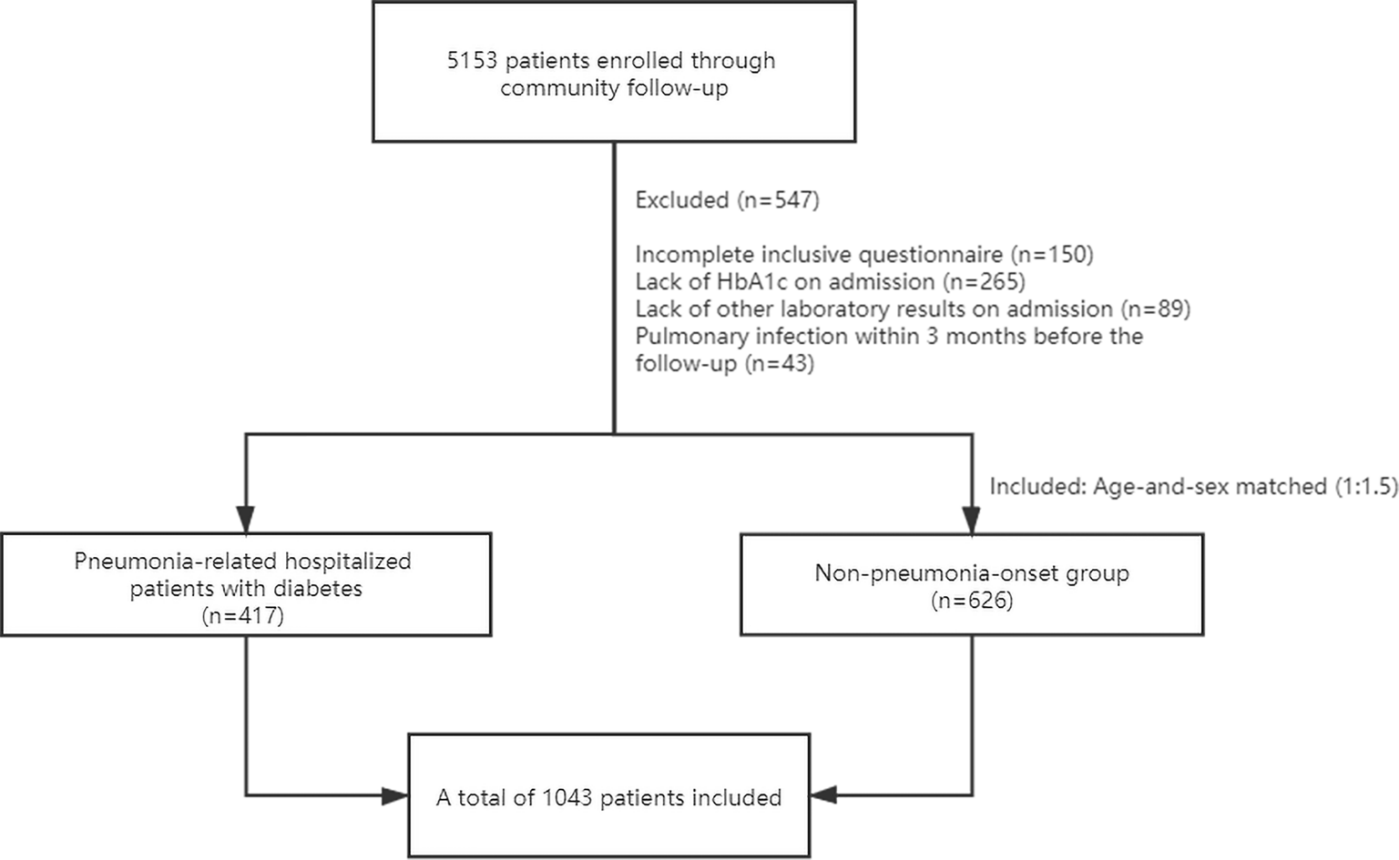
Flowchart of this study.

From 2017 to 2019, we included 417 diabetic patients with a diagnose of pneumonia. In the meantime, 626 patients with diabetes (1:1.5) who had no infection for ≥3 months prior to enrollment were included in the no-pneumonia-onset group. In addition, 43 patients without new-onset pneumonia were also excluded due to a recent diagnosis of pneumonia within 3 months (**Figure 1**).

Baseline clinical characteristics, comorbidities, and therapeutic regimens against DM are described in **Table 1**, including the comparison between the pneumonia group and the no-pneumonia-onset group. The mean age of all patients with diabetes was 67.99 years (standard deviation: 10.58), and 52.8% of the patients were males. Approximately one-third of the patients had smoking history (34.8%); among those, 12.8% had quit smoking prior to enrollment.

**Table 1:** Description of the population and comparison between patients with or without pulmonary infection.

	Total population,n = 1,043	Pneumonia-related hospitalized patients with diabetes, n = 417	Non-pneumonia-onset diabetic patients, n = 626	p-value
**Socio-demographic details**				
Age (years)	67.99 ± 10.58	68.30 ± 13.93	67.78 ± 7.40	0.43
≥75	238 (23.1)	148 (35.5)	90 (14.1)	<0.001
Male sex	551 (52.8)	254 (60.9)	297 (47.4)	<0.001
BMI (kg/m^2^)	25.15 ± 4.13	24.13 ± 4.36	25.86 ± 3.81	<0.001
<25	529 (50.7)	263 (63.1)	266 (42.5)	<0.001
Smoking				
Current smoker	230 (22.0)	92 (22.1)	138 (22.0)	0.624
Ex-smoker	135 (12.8)	59 (14.1)	76 (11.9)	
Non-smoker	678 (65.1)	266 (63.8)	412 (65.8)	
Drinking	116 (11.1)	51 (12.3)	65 (10.4)	0.333
**Comorbidity**				
CLD	174 (16.7)	95 (22.8)	79 (12.6)	<0.001
Asthma	36 (3.4)	19 (4.6)	17 (2.7)	0.093
COPD	30 (2.9)	26 (6.2)	4 (0.6)	<0.001
Bronchiectasis	21 (2.0)	15 (3.6)	6 (0.9)	0.004
Bronchitis	85 (8.1)	34 (8.2)	51 (8.0)	0.890
Tuberculosis	22 (2.1)	12 (2.9)	10 (1.6)	0.140
ILD	15 (1.4)	9 (2.1)	6 (1.0)	0.133
OSAS	3 (0.3)	3 (0.7)	0	0.061
Hypertension	407 (39.0)	244 (58.5)	163 (26.0)	<0.001
Heart failure	137 (15.1)	30 (11.2)	107 (16.8)	0.030
CHD	73 (6.9)	52 (12.5)	21 (3.3)	<0.001
Cerebral infarction	136 (13.0)	70 (16.9)	66 (10.4)	0.002
Liver cirrhosis	8 (0.8)	5 (1.2)	3 (0.5)	0.216
Renal failure	72 (6.8)	45 (10.8)	27 (4.2)	<0.001
Parkinson disease	8 (0.8)	3 (0.7)	5 (0.8)	0.838
Rheumatism	24 (2.3)	10 (2.4)	14 (2.2)	0.828
Hematopathy	18 (1.7)	17 (4.1)	1 (0.2)	<0.001
Cancer	50 (4.7)	30 (7.2)	20 (3.1)	0.002
**Characteristics of diabetes**				
DM type				
Type 1	11 (1.1)	4 (1.0)	7 (1.1)	0.463
Type 2	1,025 (98.3)	405 (98.1)	620 (98.4)	
Steroid DM	7 (0.7)	4 (1.0)	3 (0.5)	
Duration of DM (years) ≥10 years	10.94 ± 8.19 444 (42.6)	10.24 ± 8.51 196 (47.0)	11.59 ± 7.84 248 (39.6)	0.016 0.018
≥20 years ≥30 years	151 (14.5) 33 (3.2)	82 (19.7) 21 (5.0)	69 (11.0) 12 (1.9)	<0.001 0.005
≥40 years	8 (0.8)	5 (1.2)	3 (0.5)	0.278
Treatment				
Diet	226 (21.7)	60 (14.4)	166 (26.5)	<0.001
Oral agents	576 (55.2)	211 (50.6)	365 (58.3)	
Insulin	119 (11.4)	93 (22.3)	26 (4.2)	
Insulin + oral	122 (11.7)	53 (12.7)	69 (11.0)	
FBG	8.94 ± 3.68	9.64 ± 4.62	8.47 ± 2.79	<0.001
≥10 mmol/L	280 (26.5)	147 (35.3)	133 (20.8)	<0.001
PBG	12.99 ± 5.20	14.56 ± 5.71	12.38 ± 4.85	<0.001
≥13 mmol/L	403 (45.6)	146 (59.6)	257 (40.3)	<0.001
HbA1c	7.93 ± 2.20	8.42 ± 2.77	7.59 ± 1.61	<0.001
≥9%	243 (23.0)	137 (32.9)	106 (16.6)	<0.001

Continuous parameters presented as mean ± standard deviation, categorical data as n (%).

BMI, body mass index (kg/m^2^); CLD, chronic lung disease; COPD, chronic obstructive pulmonary disease; ILD, interstitial lung disease; OSAS, obstructive sleep apnea syndrome; CHD, coronary heart disease; FBG, fasting blood glucose; PBG, postprandial blood glucose; HbA1c, hemoglobin A1c.

Six hundred eighty patients (65.2%) had comorbidities with hypertension being the most commonly observed (39%), followed by chronic lung disease, heart failure, cerebral infarction, and coronary heart disease (CHD). Overall, patients with DM had more coexisting medical conditions *versus* those without diabetes, including higher prevalence of chronic obstructive pulmonary disease (COPD) (6.2% *vs*. 0.6%), bronchiectasis (3.6% *vs*. 0.9%), hypertension (58.5% *vs*. 26%), CHD (12.5% *vs*. 3.3%), cerebral infarction (16.9% *vs*. 10.4%), renal failure (10.8% *vs*. 4.2%), hematopathy (4.1% *vs*. 0.2%), and other type of malignancy (7.2% *vs*. 3.1%).

Glycemic control status and treatment of diabetes among patients in this cohort are also shown in **Table 1**. The majority of patients were diagnosed with type 2 diabetes (98%), had a long history of disease (average: 10.94 years), and elevated short- or long-term levels of blood glucose. The use ratio of insulin was notably higher in the pneumonia group regardless of the administered oral agents.

Among 417 patients with pneumonia in this study, pathogen were identified in 105 patients (25.2%). Ninety-five patients were diagnosed with bacterial infection, among which *Klebsiella pneumonia* was the most commonly isolated pathogen (27.4%, 26/95), followed by *Acinetobacter baumannii* (22.1%, 21/95), *Staphylococcus aureus* (15.8%, 15/95), *Pseudomonas aeruginosa* (11.6%, 11/95), *Streptococcus* (8.4%, 8/95), *Stenotrophomonas maltophilia* (6.3%, 6/95), and *Escherichia coli* (4.2%, 4/95); 12 patients had multi-bacterial coinfection. Meanwhile, 8 patients were diagnosed with viral infection, including influenza A and respiratory syncytial virus infection. Aspergillus was also identified in three patients.

### Risk Factors of Pneumonia

According to the above methods and analyses, the following categorical variables were entered in a backward stepwise logistic regression analysis: sex; age ≥ 75 years; body mass index (BMI) < 25; COPD; bronchiectasis; hypertension; CHD; cerebral infarction; renal failure; hemopathy; cancer; treatment of DM; use of insulin; duration of DM ≥ 20 years; HbA1c ≥ 9%; FBG ≥ 10 mmol/L; and PBG ≥ 13 mmol/L (**Table 2**).

**Table 2:** Univariate analysis associated with pulmonary infection in diabetes.

Clinical features	Univariate analysis	
	Odds ratio (95% confidence interval)	p-value
Male	1.766 (1.374–2.268)	<0.001
Age ≥75 years	3.604 (2.795–4.646)	<0.001
BMI <25 kg/m^2^	2.326 (1.806–2.994)	<0.001
CLD	2.040 (1.466–2.838)	<0.001
COPD	10.242 (3.557–29.49)	<0.001
Bronchiectasis	3.666 (1.411–9.526)	0.008
Hypertension	3.876 (2.987–5.028)	<0.001
CHD	4.096 (2.407–6.969)	<0.001
Cerebral infarction	1.772 (1.235–2.542)	0.002
Renal failure	2.652 (1.609–4.371)	<0.001
Hemopathy	23.749 (3.138–179.77)	0.002
Cancer	2.355 (1.307–4.241)	0.004
DM-treated	2.019 (1.470–2.772)	<0.001
Insulin	2.850 (2.119–3.835)	<0.001
DM ≥20 years	1.377 (0.969–1.956)	0.074
HbA1c ≥9%	2.325 (1.735–3.117)	<0.001
FBG ≥10 mmol/L	2.012 (1.520–2.662)	<0.001
PBG ≥13 mmol/L	2.159 (1.598–2.918)	<0.001

BMI, body mass index (kg/m^2^); CLD, chronic lung disease; COPD, chronic obstructive pulmonary disease; CHD, coronary heart disease; DM, diabetes mellitus; HbA1c, hemoglobin A1c; FBG, fasting blood glucose; PBG, postprandial blood glucose.

**Figure 2:**
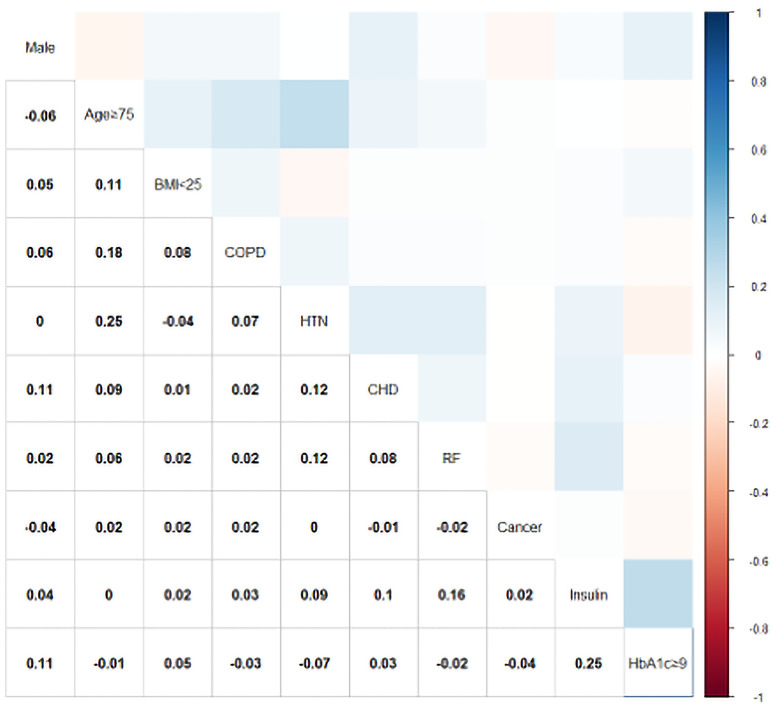
Color-coded matrices for the correlation coefficients between each predictor of the PDPI. BMI, body mass index (kg/m^2^); COPD, chronic obstructive pulmonary disease; HTN, hypertension; CHD, coronary heart disease; RF, renal failure; HbA1c, hemoglobin A1c.

Relative weights were assigned according to the regression coefficient of each categorical variable (β) to develop a simple and useful clinical prediction tool. The following parameters were included as predictive factors: male sex (OR = 1.72, 95% CI: 1.27–2.33, p < 0.001), age ≥ 75 years (OR = 2.31, 95% CI: 1.61–3.31, p < 0.001), BMI < 25 (OR = 2.59, 95% CI: 1.92–3.50, p < 0.001), COPD (OR = 6.58, 95% CI: 2.09–20.7, p = 0.001), hypertension (OR = 4.27, 95% CI: 3.12–5.85, p < 0.001), CHD (OR = 2.98, 95% CI: 1.61–5.52, p < 0.001), renal failure (OR = 1.82, 95% CI: 1.002–3.29, p = 0.049), cancer (OR = 3.57, 95% CI: 1.80–7.06, p < 0.001), use of insulin (OR = 2.28, 95% CI: 1.60–3.25, p < 0.001), and HbA1c ≥ 9% (OR = 2.70, 95% CI: 1.89–3.85, p < 0.001). The VIF of each variable was <2.0. The results of the correlation analysis are shown in **Figure 2**; all coefficients of correlation were <0.3, indicating a weak correlation between any each two predictors.

### Prognostic Index and Risk Groups

The prognostic score was named PDPI (pneumonia in diabetic patients predictive index) and calculated as follows:

$\begin{array}{c} \text{PDPI}\;\text{score}\;=\;28.806\;\left(\text{if}\;\text{male}\right)\\ +\;44.442\;\left(\text{if}\;\text{age}\;\geq \;75\;\text{years}\right)\\ +\;50.53188\;\left(\text{if}\;\text{BMI}\;<\;25\;\text{kg}/{\text{m}}^{2}\right)\\ +\;52.66696\;\left(\text{if}\;\text{HbA}1\text{c}\;\geq \;9\%\right)\\ +\;31.64817\;\left(\text{if}\;\text{has}\;\text{renal}\;\text{insufficiency}\right)\\ +\;100\;\left(\text{if}\;\text{COPD}\right)\\ +\;77.05523\;\left(\text{if}\;\text{hypertension}\right)\\ +\;58.0313\;\left(\text{if}\;\text{CHD}\right)\\ +\;67.4955\;\left(\text{if}\;\text{cancer}\right)\\ +\;43.83994\;\left(\text{if}\;\text{use}\;\text{insulin}\;\text{as}\;\text{treatment}\;\text{of}\;\text{diabetes}\right) \end{array}$

**Figure 3:**
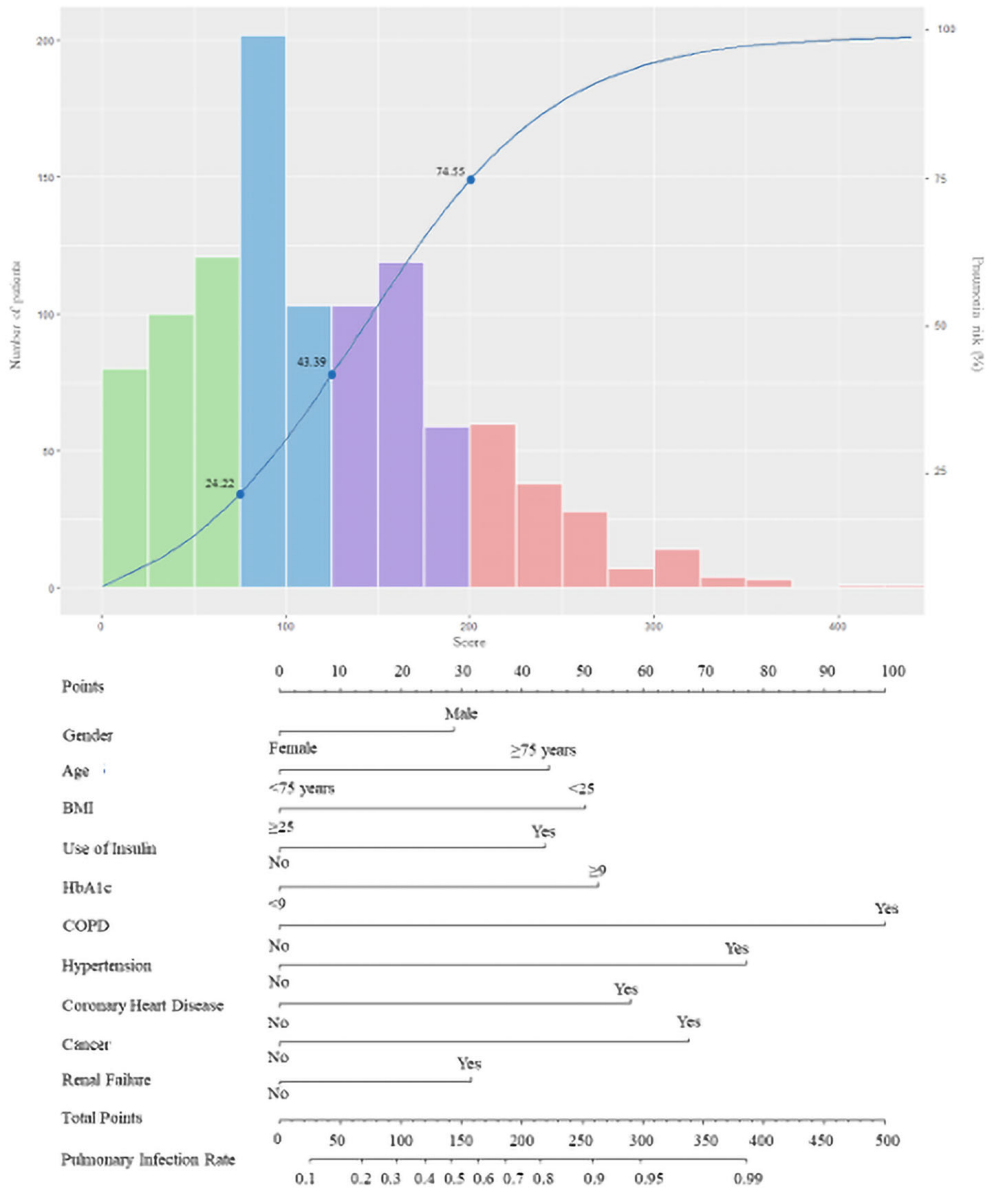
The score nomogram for bedside application. The blue curve refers to the risk of pneumonia in patients with diabetes. The histogram refers to the distribution of the PDPI score in the derivation cohort: green bars, score < 75 (low risk); blue bars, 75 ≤ score < 125 (moderate risk); purple bars, 125 ≤ score < 200 (high risk); and red bars, score ≥ 200 (markedly high risk). Each axe below corresponds to the predicting score of the PDPI, while the two axes on the bottom reflect the relationship between the total score and the probability of pneumonia.

Accordingly, the rate of pneumonia in patients with diabetes was shown in nomogram (**Figure 3**).

The median value of the prognostic score in 1,043 patients was 115.6 (range: 0–443.8), 10% of the values were <28.8, and 90% of the values were <224.1. Potential cut-off points between scores 25 and 225 in steps of 25 were assessed. On the basis of this approach, the value of 75 (C-statistic: 0.657; sensitivity: 0.899; specificity: 0.414) was considered as the cut-off point of the low-risk group, which included 301 patients (28.9%; score < 75; risk < 0.2422). Overall, the value of 125 showed the best discrimination (C-statistic: 0.748; sensitivity: 0.717; specificity: 0.780) and defined the moderate-risk group, which included 305 patients (29.2%; 75 ≤ score < 125; 0.2444 ≤ risk < 0.4339). For the definition of the high-risk group, the value of 200 was identified as the best discriminator (C-statistic: 0.603; sensitivity: 0.317; specificity: 0.962). Accordingly, 281 patients were classified in the high-risk group (26.9%; 125 ≤ score < 200; 0.4339 ≤ risk < 0.7455). The remaining 156 patients belonged to the markedly high-risk group (15.0%; score > 200; risk ≥ 0.7455). After Bonferroni correction for the p-values calculated using the C-statistics, the separation of these risk groups remained statistically significant. The characteristics of patients classified in the different risk groups based on the predictive score are reported in **Table 3**.

**Table 3:** Patient characteristics according to the risk categories determined using the predictive score.

	Total score				
Characteristics	<75	75–125	125–200	≥200	p-value
N (%)	301 (28.9)	305 (29.2)	281 (26.9)	156 (15.0)	
Pneumonia	42 (14.0)	76 (24.9)	167 (59.4)	132 (84.6)	<0.001
Age (years)	65.70 ± 7.93	66.62 ± 9.75	68.42 ± 11.45	75.32 ± 10.97	<0.001
≥75	21 (7.0)	44 (14.4)	81 (28.8)	92 (59.0)	<0.001
Male sex	94 (31.2)	176 (57.7)	169 (60.1)	112 (71.8)	<0.001
BMI (kg/m^2^)	26.56 ± 4.06	25.70 ± 3.69	24.33 ± 3.97	23.04 ± 4.13	<0.001
<25	77 (25.6)	145 (47.5)	182 (64.8)	125 (80.1)	<0.001
Smoking					
Current smoker	41 (13.6)	91 (29.8)	66 (23.5)	31 (20.0)	<0.001
Ex-smoker	34 (11.3)	29 (9.5)	43 (15.3)	29 (18.7)	
Non-smoker	226 (75.1)	185 (60.7)	172 (61.2)	95 (61.3)	
Chronic lung disease	42 (14.0)	42 (13.8)	46 (16.4)	44 (28.2)	<0.001
COPD	0	0	7 (2.5)	23 (14.7)	<0.001
Hypertension	0	108 (35.4)	159 (56.6)	140 (89.7)	<0.001
Coronary heart disease	1 (0.3)	5 (2.3)	20 (7.1)	44 (28.2)	<0.001
Renal failure	6 (2.0)	10 (3.3)	26 (9.3)	30 (19.2)	<0.001
Cancer	6 (2.0)	7 (2.3)	21 (7.5)	15 (9.6)	<0.001
Duration of DM (years)	10.11 ± 7.88	10.13 ± 7.42	11.08 ± 8.24	13.60 ± 9.39	<0.001
Treatment					
Diet	86 (28.6)	101 (33.1)	25 (8.9)	14 (9.0)	<0.001
Oral agents	190 (63.1)	167 (54.8)	160 (56.9)	59 (37.8)	
Insulin	9 (3.0)	15 (4.9)	53 (18.9)	42 (26.9)	
Insulin + oral agents	16 (5.3)	22 (7.2)	43 (15.3)	41 (26.3)	
FBG	7.97 ± 2.38	8.92 ± 3.12	9.24 ± 4.31	10.27 ± 4.73	<0.001
≥10 mmol/L	48 (15.9)	83 (27.2)	86 (30.6)	62 (39.7)	<0.001
PBG	11.84 ± 4.18	12.90 ± 5.11	13.92 ± 5.93	14.90 ± 5.19	<0.001
≥13 mmol/L	105 (36.5)	121 (44.2)	109 (52.2)	66 (66.0)	<0.001
HbA1c	7.16 ± 1.16	7.61 ± 1.64	8.52 ± 2.29	9.00 ± 3.45	<0.001
≥9%	14 (4.7)	51 (16.7)	110 (39.1)	67 (42.9)	<0.001

BMI, body mass index (kg/m^2^); COPD, chronic obstructive pulmonary disease; DM, diabetes mellitus; FBG, fasting blood glucose; PBG, postprandial blood glucose; HbA1c, hemoglobin A1c.

### Validation and Performance of Nomogram

**Figure 4:**
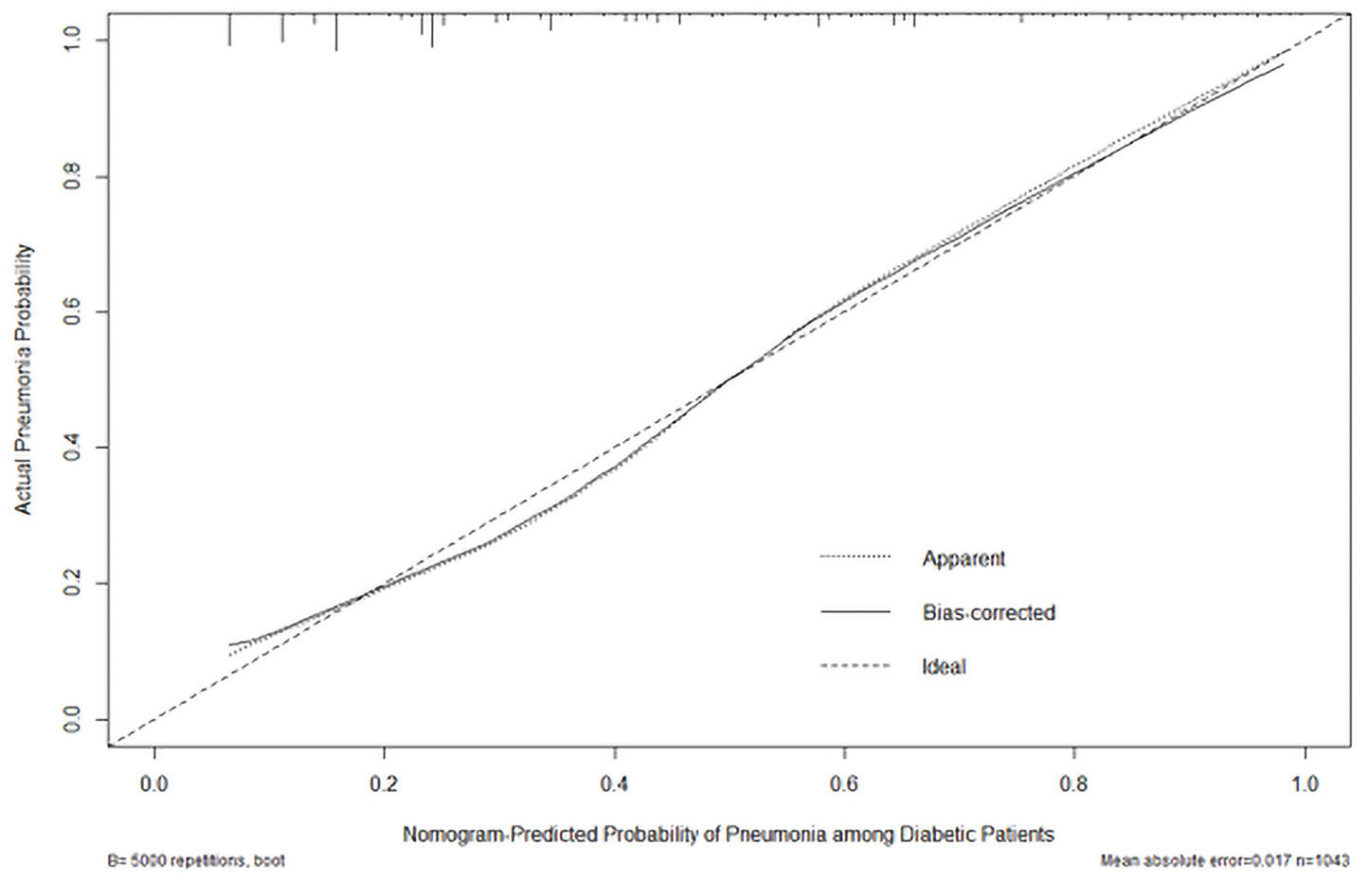
Calibration plot of the actual risk probability (y-axis) over the predicted risk probability (x-axis) based on the nomogram of PDPI in **Figure 3**.

For the total study population (1,043 patients), the C-statistics value for the nomogram was 0.811 (95% CI: 0.784–0.838). The sensitivity and specificity of this model were 0.717 and 0.780 under cut-off of 125 score. The AUROC of the training set was 0.821 (95% CI: 0.791–0.851), while that of the testing set was 0.771 (95% CI: 0.705–0.836), respectively. The Hosmer–Lemeshow type χ^2^ statistics value was 13.637 (p = 0.092), indicating a good fit for the model. The calibration curve of the score using 5,000 bootstrapped dataset is shown in **Figure 4**, confirming a satisfactory accuracy. The validation was also performed using 10-fold cross-validation in the derivation cohort, which revealed an accuracy of 0.7788 (95% CI 0.7287–0.8237, p = 2.26 × 10^−5^).

## Discussion

We developed the PDPI based on 1,043 patients with diabetes from our population-based, prospective cohort study conducted in China. To our knowledge, this is the first predictive tool specifically aiming at the prediction of the risk of pneumonia among diabetic patients. Moreover, it allows the discrimination of four risk groups of patients with significantly different stages of pneumonia based on 10 independent risk factors (i.e., sex, age, BMI, use of insulin, HbA1c, COPD, hypertension, CHD, renal failure, and cancer), which are easy-to-acquire variables in clinical practice.

In our study, patients with DM were significantly older than 75 years and most of them were male (both p < 0.001); thus, we did not match the age and sex of the two groups of patients as these could be potential factors. Patients with confirmed DM and pneumonia were even older (mean age: 74.36 years), as previously observed in a retrospective study (Bader et al., 2016). Of note, there was a general male predominance (range: 52.9–64.8%) in diabetic patients with pneumonia (Huang et al., 2015; Falcone et al., 2016; Lopez-de-Andres et al., 2017). However, we may have underestimated the importance of sex due to the common adjustment in case-control studies. Males were prone to infections and malignancies probably owing to the lack of estrogen, which could modulate different immune functions, and the single X chromosome, on which several genes encoding innate immune molecules are located (Jaillon et al., 2019). As expected, the logistic regression model utilized in our study showed that the correlation between the risk of pneumonia and the two variables was significant.

Our data confirmed and extended those of previous studies (Kornum et al., 2008; Hine et al., 2017) suggesting that poor glycemic control and longer duration of diabetes may increase the risk of pneumonia. Studies regarding the effects of chronic hyperglycemia on the onset of pneumonia were rather affirmative. Poorer chronic glycemic control was associated with increased risk of pneumonia-related hospitalization, ventilator-associated pneumonia, and septicemia (Tsakiridou et al., 2013). Consistent with the study conducted by Kornum et al. (2008), we found that patients with higher levels of HbA1c (i.e., ≥9%) were at increased risk of pneumonia. The American Diabetes Association suggested a biannual HbA1c test in patients meeting treatment goals and achieving stable glycemic control; a quarterly test was recommended for patients who changed therapy or not meeting glycemic goals (American Diabetes Association, 2018a). Therefore, a routine HbA1c test may support the timely treatment of diabetes and prevention of acute infectious diseases.

Meanwhile, short-term levels of glucose with a cut-off value of 180 mg/dl (10 mmol/L) was independently associated with pneumonia when other risk factors were controlled (Jeon et al., 2012). This was also confirmed by another case-control cohort study showing that patients with diabetes hospitalized due to pneumonia had higher levels of glucose (MacIntyre et al., 2012). The correlation between the short-term levels of glucose and status of pneumonia was biologically plausible, given that acute hyperglycemia may alter cellular defenses by affecting neutrophil chemotaxis, phagocytosis, respiratory burst, and antimicrobial activity (Jafar et al., 2016). Our study also revealed a relationship between short-term hyperglycemia >10 mmol/L and the onset of pneumonia. More than half (51.1%) of our 280 patients with FBG ≥ 10 mmol/L were diagnosed with pneumonia. However, the FBG levels for the pneumonia group were determined on admission. Meanwhile, more than half of the patients were reported to have stress-induced hyperglycemia, including those without diabetes or individuals with undiagnosed diabetes (Kerby et al., 2012; Chen et al., 2015). These also shed a light on the importance of FBG in surveillance.

We also found a difference in risk estimates for pneumonia according to the duration of diabetes (increase of 10 years). This finding was consistent with the data reported in a study conducted by Koziel and Koziel (1995). Notably, longer duration may cause progressive microangiopathies in the basement membranes of pulmonary blood vessels and respiratory epithelium. However, this was not included as an independent predictor. Regarding treatment, the use of insulin was a risk factor for pneumonia in our study. Although insulin was reported to exert anti-inflammatory effects in patients with moderate degree of hyperglycemia (Das, 2001), it was simultaneously associated with refractory poor glucose control and was not protective against hospitalization due to infection (Hamilton et al., 2013).

In addition to hyperglycemia, several comorbidities played a prominent role in pneumonia by influencing the spectrum of causative agents and facilitating the growth of unusual microorganisms (McDonald et al., 2015; Gustinetti and Mikulska, 2016). Endothelial dysfunction in patients with diabetes was regarded as one of the major factors in the development of cardiovascular diseases, such as hypertension and CHD (Schiekofer et al., 2000). Meanwhile, renal dysfunction is widely established as a complication of diabetes. Microangiopathies, such as nephropathy and ischemic heart disease, have been independently associated with increased risk of bacterial infection (Hamilton et al., 2013), which was consistent with our result. The relationship between renal failure, cardiovascular disease, and infection is complex. In a retrospective cohort study, the association of chronic kidney disease with pneumonia appeared to be partly due to underlying accrued cardiovascular and cerebrovascular comorbidities; a strong graded association remained after adjustment for these, suggesting chronic kidney disease as a surrogate risk marker (McDonald et al., 2015).

For clinicians and policy makers, it is essential to take the comorbidities into account. Clinical trials investigating the value of treating hypertension in older patients with diabetes have highlighted the importance of controlling cardiovascular factors, instead of focusing on tight glycemic control alone (Beckett et al., 2008; American Diabetes Association, 2018b). Meanwhile, we should pay attention to the surveillance of renal function, including estimated glomerular filtration rate and proteinuria for health service planning (Go et al., 2004).

Immune dysfunction in diabetes has been recognized as one of the possible etiological factors of pneumonia (Yende et al., 2010). Several comorbidities altering the immune function may contribute to this process. A previous study concluded that non-diabetic patients with any type of malignancy had longer survival than other non-diabetic patients without cancer (Lopez-de-Andres et al., 2017), suggesting a relationship between cancer and pneumonia. In this study, we also found that cancer was more prevalent in diabetic patients with pneumonia. Furthermore, the randomized controlled trials reported an increased risk of pneumonia among outpatient populations with COPD, in whom autoimmune and inflammatory processes were the most important drivers (Hirano et al., 2018). A mouse model of COPD showed a change in the T cell receptor repertoire suggestive of an antigen-specific response, which would lead to alveolar destruction and inflammation (Eppert et al., 2013). Moreover, a previous study showed that pre-existing diabetes was associated with higher pneumonia-related mortality due to worsening of cardiovascular and kidney diseases (Yende et al., 2010). Since the mechanisms underlying the increased risk of pneumonia among diabetic patients remain unclear, larger-scale studies are warranted to further investigate the immune responses against different etiological agents.

Diabetes is commonly associated with a higher BMI compared with that reported in non-diabetics. Nevertheless, the mechanism through which BMI acts as a predictive factor of pneumonia remains controversial. Hamilton et al. found that a higher BMI (increase of 1 kg/m^2^) was associated with hospitalization for any infection (Hamilton et al., 2013). Furthermore, research on diabetic patients showed that lower BMI may further indicate acute infectious illness, especially community-acquired pneumonia caused by *Klebsiella pneumoniae (* Huang et al., 2015). In our study, we found that a larger number of diabetic patients with a BMI <25 kg/m^2^ acquired pneumonia. Emaciation of the body was hypothesized to be a reflection of general reduction in vitality and poor glucose control. Hence, the inverse association between low BMI and new onset of pneumonia may indicate a tendency toward infection in patients with poor health condition.

The main strength of our study was its population-based, prospective, cohort design. By collecting data on potential confounding factors prior to the onset of pneumonia, we were able to avoid the recall bias based on consultations after admission. Our simple score would be practical with the extension of the mobile application in the future.

Some limitations should also be acknowledged. The sample size was relatively small to establish a predictive score. Despite the limitation, the study was designed to reflect the ‘real life’ clinical situation. Clinical information was meticulously gathered using standard protocols by admitted medical team.

## Conclusion

Our new, easy-to-use, clinically predictive, classification tool may be helpful in assessing the risk of pneumonia among patients with diabetes. This score emphasizes the importance of glycemic control, regular follow-up of chronic diseases, and targeted immunization for the purpose of early prevention. These results allow the stratification of diabetic patients into different relevant risk categories and provide a basis for individually tailored intervention.

## Data Availability Statement

The raw data supporting the conclusions of this article will be made available by the corresponding author by request.

## Ethics Statement

This study was approved by the Coordinating Ethics Committee of Ruijin Hospital Affiliated to Shanghai Jiao Tong University School of Medicine (No.2017-205) and registered in the clinicaltrials.gov database (NCT 03617393). The patients/participants provided their written informed consent to participate in this study.

## Author Contributions

Conception or design of the work: MZ and JQ. Data collection: LG, NL, BQ, BH, HY, JH, and BL. Data analysis and interpretation: LG and YS. Draft of the article: LG. Critical revision of the article: YH, YS, MZ, and JQ. All authors contributed to the article and approved the submitted version.

## Funding

This work was supported by the National Key R&D Program of China (Grant Nos. 2017YFC1309700 and 2017YFC1309701), by Shanghai Top-Priority Clinical Key Disciplines Construction Project (Grant No. 2017ZZ02014) and by Shanghai Municipal Key Clinical Specialty (Grant No. shslczdzk02202). This work was also funded in part by a grant from Innovative research team of high-level local universities in Shanghai, by Shanghai Key Laboratory of Emergency Prevention, Diagnosis and Treatment of Respiratory Infectious Diseases (Grant No. 20dz2261100) and by Cultivation Project of Shanghai Major Infectious Disease Research Base (Grant No. 20dz2210500).

## Conflict of Interest

The authors declare that the research was conducted in the absence of any commercial or financial relationships that could be construed as a potential conflict of interest.

## Publisher’s Note

All claims expressed in this article are solely those of the authors and do not necessarily represent those of their affiliated organizations, or those of the publisher, the editors and the reviewers. Any product that may be evaluated in this article, or claim that may be made by its manufacturer, is not guaranteed or endorsed by the publisher.

## Abbreviation

OR, odds ratio; CI, confidence interval; DM , diabetes mellitus; VIF, variance inflation factor; AUROC, area under the receiver operating characteristic curve; BMI, body mass index (kg/m2); CLD, chronic lung disease; COPD, chronic obstructive pulmonary disease; ILD, interstitial lung disease; OSAS, obstructive sleep apnea syndrome; CHD, coronary heart disease; FBG, fasting blood glucose; PBG, postprandial blood glucose; HbA1c, hemoglobin A1c; PDPI, pneumonia in diabetic patients predictive index.
